# Synergistic effects of defocus-incorporated multiple segments and atropine in slowing the progression of myopia

**DOI:** 10.1038/s41598-022-25599-z

**Published:** 2022-12-24

**Authors:** Zhu Huang, Xu-Fei Chen, Ting He, Yun Tang, Chi-Xin Du

**Affiliations:** 1 Department of Ophthalmology, The First Affiliated Hospital, College of Medicine, Zhejiang University, Hangzhou, 310003 China; 2 Department of Ophthalmology, The Fourth Affiliated Hospital, School of Medicine, Zhejiang University, Yiwu, 322000 China

**Keywords:** Diseases, Health care

## Abstract

Myopia is a leading cause of visual impairment in young people worldwide. It sometimes increases the risk of blindness and reduces life quality. Previous reports have revealed the treatment effects of defocus-incorporated multiple segments (DIMS) and topical atropine (ATP) on myopia control. However, no study has evaluated these two interventions together. In this retrospective study, we aimed to determine whether the combination of DIMS lenses and 0.01% ATP can slow the progression of myopia compared with DIMS lenses or single vision (SV) lenses alone. We included 107 children with myopia who were treated with DIMS and 0.01% ATP combination (DIMS + ATP group), DIMS monotherapy (DIMS group), or a control group (SV group). We compared treatment effects among three groups in axial length and myopia progression. After a 1-year follow-up, the DIMS + ATP group showed a smaller change in axial length and myopia progression than the DIMS and SV groups (P < 0.05). Hence, combination treatment with DIMS and 0.01% ATP might be a better choice for children with myopia.

## Introduction

Myopia is one of the leading causes of visual impairment in young people worldwide^1,2^. The prevalence of myopia in children and adolescents has rapidly increased in recent decades, particularly in East Asia^3–5^. A study in school-aged children from Wuhan City in central China revealed that the prevalence of myopia remains less than 7% at the age of 5 years but more than 50% at the age of 7 years and more than 85% at the age of 16 years^3^. Myopia in childhood can easily progress to high myopia in adulthood, increasing the risk of retinal detachment, macular neovascularization, cataract, and glaucoma that can result in significant visual impairment or blindness^6–9^. Therefore, prevention and intervention with regard to myopia at school age are of paramount importance.

Currently, several effective optical methods, including orthokeratology lenses^10–14^, dual-focus soft contact lenses^7^, peripheral defocus-modifying spectacle lenses^15^, and other less popular methods, are available for slowing the progression of myopia in children. Their efficacy for myopia control ranges from 30 to 60%^15^. The defocus-incorporated multiple segment (DIMS) spectacle^16–19^ is a new style of peripheral defocus-modifying spectacle lenses, which contain a central optical zone for correcting distance refractive errors to clear distance eyesight and an annular multiple focus zone for introducing myopic defocus (MD) to control myopia progression. According to a 2-year double-masked randomized controlled experiment, myopia progression was 52% slower in the DIMS group than in the single vision (SV) group, and axial elongation was 62% less in the DIMS group than in the SV group^18^.

Moreover, pharmaceutical agent interventions for myopia include the administration of atropine (ATP)^15^, pirenzepine^20^, and 7-methylxanthine^21^, which have shown promising results in early clinical trials. ATP, a nonselective antimuscarinic agent, is the only pharmaceutical agent that has been broadly adopted for myopia control for decades. The efficacy of ATP^22^ and several adverse effects, such as the rebound effect^22^, pupillary diameter, and accommodative amplitude, are dose-dependent^23^. Thus, low-concentration ATP may be a safe dose for slowing myopia progression in children with the least side effect.

DIMS is generally thought to decrease the advancement of myopia by an optical mechanism, whereas ATP slows myopia progression through a pharmacological mechanism. However, combining therapies with diverse mechanisms of action may be more beneficial than monotherapy in reducing myopia progression. In the present study, we compared myopia progression with DIMS lenses plus 0.01% ATP to DIMS lenses or SV lenses alone.

## Materials and methods

### Patients

**Figure 1:**
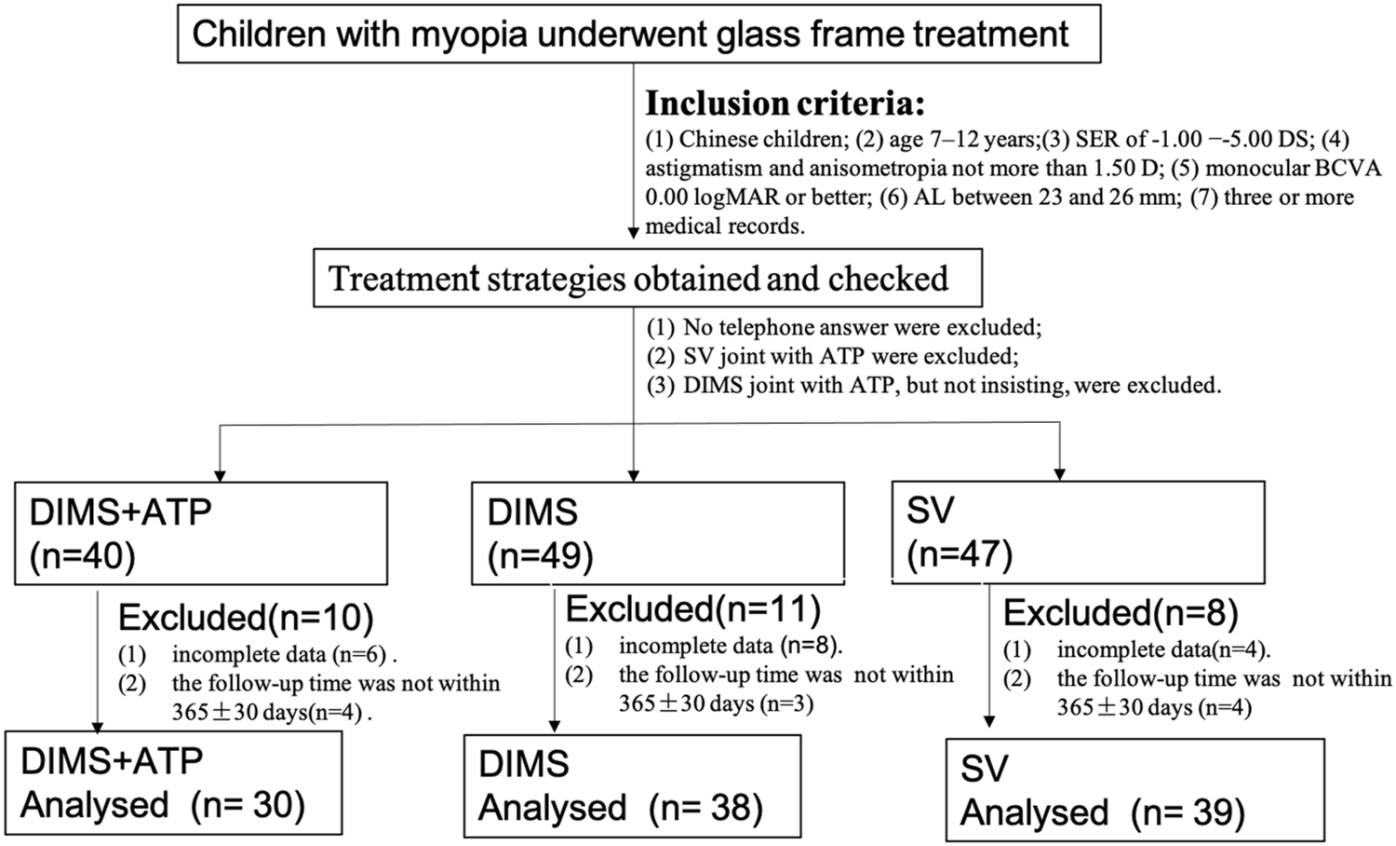
Schematic diagram of patient selection and study design. *ATP* atropine, *AL* axial length, *BCVA* best corrected visual acuity, *D* diopter, *DIMS* defocus-incorporated multiple segments, *DS* diopter sphere, *SER* spherical equivalent refraction.

In this retrospective cohort study, the medical records of children with myopia who underwent spectacle lens treatment at The Fourth Affiliated Hospital of Zhejiang University (Yiwu, Zhejiang) from June 2020 to December 2021 were reviewed. The treatment strategies were obtained from the hospital information system and checked whether the treatment strategies were executed for the children as required and according to the purchase records of the glass frames from the glasses sales department. Parents were called on the phone to confirm whether they simultaneously used atropine eye drops for the children. Only three treatment strategies including a combination of 0.01% ATP and DIMS lenses simultaneously, DIMS lenses alone or SV lenses alone were analysed in this study. In total, 136 children with myopia met the criteria for the study. Due to incomplete data for 18 patients, the follow-up time not within 365 ± 30 days for 11 patient, finally 107 children with myopia who underwent treatment with a combination of 0.01% ATP and DIMS lenses, DIMS lenses alone or SV lenses alone were identified and used in our statistical analysis. Figure 1 depicts the study flow. This study adhered to the tenets of the Declaration of Helsinki and was approved by the ethics committee of The Fourth Affiliated Hospital of Zhejiang University, and a waiver for informed consent was obtained for the study from the ethics committee of The Fourth Affiliated Hospital of Zhejiang University.

### Inclusion and exclusion criteria

Inclusion criteria were as follows: (1) Chinese children; (2) age 7–12 years; (3) a spherical equivalent refractive error (SER) of − 1.00 to − 5.00 diopter sphere (DS); (4) astigmatism and anisometropia not more than 1.50 D; (5) monocular best corrected visual acuity (BCVA) 0.00 logMAR or better; (6) baseline axial length (AL) between 23 and 26 mm; and (7) three or more medical records. Exclusion criteria were as follows: (1) strabismus and binocular vision abnormalities; (2) ocular and systemic abnormalities; (3) the history of myopia control; (4) incomplete data; and (5) the follow-up time was not within 365 ± 30 days.

### DIMS lens

The DIMS lens, also known as Hoya MiyoSmart (Hoya Corporation), is a plastic spectacle lens. It consists of a central optical zone (9 mm in diameter) for correcting distance refractive errors and an annular multiple focus zone with numerous segments (33 mm in diameter) with a relative positive power (+ 3.50 D) for introducing MD. Each segment has a diameter of 1.03 mm. This design provides the wearer with clear vision at all viewing distances while incorporating MD to help control myopia progression. Multiple foci from MD are present in a plane in front of the retina, resulting in blurred retinal images.

### ATP eye drops

Low-dose ATP eye drops (Myopine, 0.01% ATP sulfate, 0.1 mg/1 mL, 0.4 mL/vial) were brought from Shenyang Xingqi Pharmaceutical Co., Ltd. (Shenyang, Liaoning). Since atropine eye drops are unavailable in our hospital, external prescriptions were issued according to the children’s intraocular pressure, angle opening, and myopia progress. Parents bought atropine eye drops produced by Shenyang Xingqi Pharmaceutical from Shenyang Xingqi Online Eye Hospital according to the doctor’s prescription.

### Measurements and follow‐up

At the first visit, screening tests were performed under full cycloplegia by instillation of four drops of 1% tropicamide for 35 min. A series of comprehensive ocular health examinations, including cycloplegic autorefraction, measured using an auto-kerato-refractometer (ARK 1s; Nidek, Japan), was performed. Furthermore, corneal keratometry and AL data were obtained using optical ocular biometry (Lenstar LS 900; Haag-Streit Diagnostics, Koeniz, Switzerland). Each parameter was set using the mean of three consecutive measurements.

The routine follow-up time was set at 6 months after lens wear. Spectacle-corrected VA, SER error, corneal power, keratometry, and AL were investigated during each visit. If myopia progression was greater than 0.5 D, the lens was replaced.

Two main results as indicators for the treatment were obtained. They were the changes in SER (ΔSER/year) and in AL (ΔAL/year). Their definitions can be found in Eqs. (1) and (2) as follows:

1 $$ {\text{Change in SER}}: \, \Delta {\text{SER}}/{\text{year}} = \left( {A - B} \right) \times {365}/{\text{follow-up time }}\left( {\text{d}} \right), $$

A: SER at baseline; B: SER at 1-year follow-up.

2 $$ {\text{Change in AL}}: \, \Delta {\text{AL}}/{\text{year}} = \left( {A - B} \right) \times {365}/{\text{follow-up time }}\left( {\text{d}} \right), $$

A: AL at baseline; B: AL at 1-year follow-up.

Z.H. and T.H obtained the treatment strategies from the hospital information system and checked whether the treatment strategies were executed for the children as required and according to the purchase records of the glass frames from the glasses sales department. Parents were called to confirm whether they simultaneously used atropine eye drops for the children. Z.H. and T.H manually exported age, SER, and sex data from Hospital Information System and checked them together. Y.T. and X.F.C. exported data on AL, K1, and K2 from Lenstar LS 900 machine and checked them together.

### Statistical analysis

Based on their characteristics, data are described as frequencies, means ± SD, and proportions (median, minimum, and maximum). Statistics were performed for differences in all groups. The chi-square and Fisher’s exact tests were adopted to evaluate categorical variables. One-way analysis of variance, or the Kruskal–Wallis test, was used to analyze the significant differences in groups. Post-hoc tests were carried out by LSD correction. Furthermore, the associations between risk factors and ΔAL in the right eye were evaluated by multiple linear regression in the follow-up period. The covariates in the multiple linear regression model include baseline AL, treatment, intervention age, sex, and baseline SER. Significant statistical difference was defined when P < 0.05. All statistical tests were performed by the software SPSS Statistics for Windows, version 19.0 (SPSS Inc., Chicago, IL, USA).

## Results

### Subjects and their characteristics

The study included 107 children with certain inclusion and exclusion criteria between June 1, 2020, and December 31, 2021. These children were divided into different groups based on the treatment options of DIMS and 0.01% ATP combination (combination group), DIMS monotherapy (DIMS group), and a control group (SV group). Table 1 presents the patients’ demographic data. The average intervention age was 9.06 ± 0.99 years, and the mean proportion of male patients was 53.3%. The average initial SER was 2.59 ± 1.11 diopter (D), and the average initial AL was 24.66 ± 0.82 mm. Age, sex, SER, AL, flat keratometry, and steep keratometry were balanced among the three groups. There were no significant differences among them (*P* > 0.05).

**Table 1:** Demographics (N = 107).

	Total	DIMS + ATP (n = 30)	DIMS (n = 38)	SV (n = 39)	*P*
Age (years)	9.06 ± 0.99	9.00 ± 0.90	8.93 ± 0.97	9.22 ± 1.08	0.419*
Sex (male)	57 (53.3)	14(46.7)	19 (50.0)	24 (61.5)	0.415^†^
Female	50(46.7)	16 (53.3)	19 (50.0)	15 (38.5)	
SER (D)	2.59 ± 1.11	2.71 ± 1.11	2.48 ± 1.24	2.60 ± 0.99	0.687*
AL (mm)	24.66 ± 0.82	24.79 ± 0.92	24.62 ± 0.87	24.60 ± 0.71	0.580*
K1 (D)	42.74 ± 1.36	42.70 ± 1.67	42.63 ± 1.37	42.88 ± 1.07	0.706*
K2 (D)	43.95 ± 1.63	43.91 ± 2.00	43.95 ± 1.50	43.96 ± 1.47	0.991*

Data are presented as n (%) or mean ± standard deviation.

*ATP* atropine, *AL* axial length, *D* diopter, *DIMS* defocus-incorporated multiple segments, *K1* flat keratometry, *k2* steep keratometry, *SER* spherical equivalent refraction.

P < 0.05 was considered statistically significant.

*ANOVA test.

^†^Chi-square test.

### Changes in measurements

**Figure 2:**
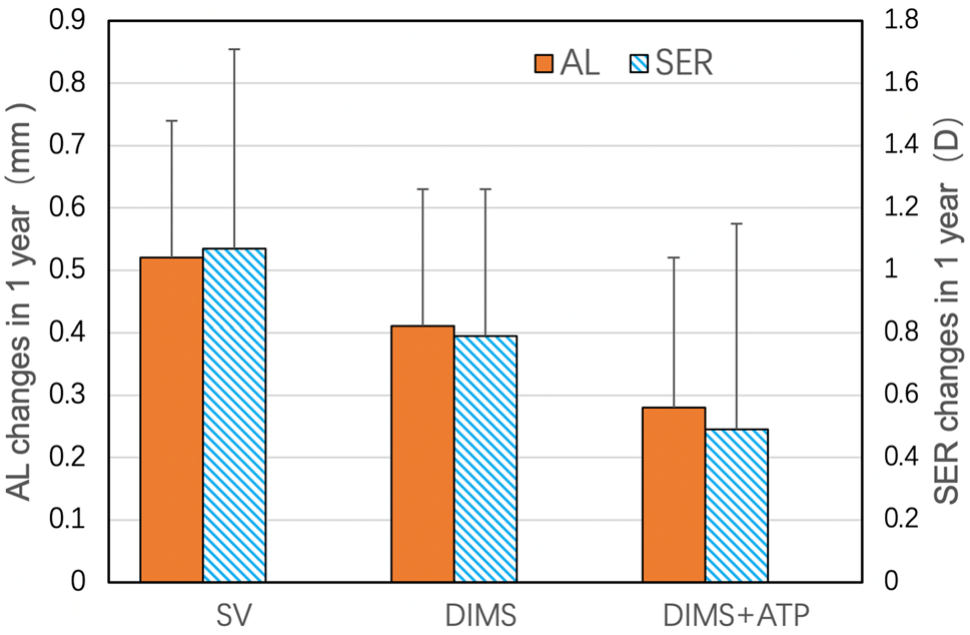
Changes in axial length and spherical equivalent refraction over 1 year in the DIMS and 0.01% ATP combination group, DIMS monotherapy group, and SV group using ANOVA test.

Changes in AL and SER were compared among the three groups (Table 2, Fig. 2). Statistical analysis showed that the AL over 1 year increased by 0.28 ± 0.24 mm in the combination group, 0.41 ± 0.22 mm in the DIMS group, and 0.52 ± 0.22 mm in the SV group. Significant differences were found between the three groups (*P* < 0.001, ANOVA test). Statistical analysis also showed that the myopia progression over 1 year increased by 0.49 ± 0.66 D in the combination group, 0.79 ± 0.47 D in the DIMS group, and 1.07 ± 0.64 D in the SV group. Significant differences were also noted between the three groups (*P* < 0.001, ANOVA test). The results of the post-hoc exploratory analyses were as follows.

**Table 2:** Changes in axial length and spherical equivalent refraction over 1 year in the DIMS and 0.01% ATP combination group, DIMS monotherapy group, and SV group using ANOVA test.

	Total	Group A DIMS + ATP	Group B DIMS	Group C SV	P value	Post-hoc
AL (mm)	0.41 ± 0.24	0.28 ± 0.24**^, #^	0.41 ± 0.22*	0.52 ± 0.22	< 0.001	B,C > A, C > B
95% CI	(0.37–0.46)	(0.19–0.37)	(0.34–0.48)	(0.45–0.60)		
SER (D)	0.81 ± 0.61	0.49 ± 0.66**^, #^	0.79 ± 0.47*	1.07 ± 0.64	0.001	B,C > A, C > B
95% CI	(0.69–0.93)	(0.24–0.74)	(0.64–0.95)	(0.86–1.28)		

*ATP* atropine, *AL* axial length, *CI* confidence interval, *D* diopter, *DIMS* defocus-incorporated multiple segments, *SER* spherical equivalent refraction.

Data are expressed as the mean ± standard deviation.

*Compared with the SV group, P < 0.05, **P < 0.01, ^#^compared with the DIMS group, P < 0.05. Post-hoc tests were performed using the LSD correction.

### Factors associated with AL and SER changes

Table 3 presents some factors and their associations with changes in AL and SER. Variable factors, including the different treatment modalities, age at intervention, sex, baseline SER, and baseline AL were calculated for their correlation to AL change with a multivariate linear regression test. The data showed no significant effect on AL change (*P* = 0.740, 0.859, and 0.833, respectively) among sex, baseline SER, and baseline AL groups, whereas age at intervention had a significant effect on AL change (*P* = 0.003). Among the treatment modalities, both DIMS + ATP and DIMS showed a significant difference in AL change when compared to SV (DIMS + ATP, β =  − 0.262, *P* < 0.001, DIMS, β =  − 0.135, *P* = 0.009). We then used variable factors of the different treatment modalities, age at intervention, sex, baseline SER, and baseline AL to calculate each correlation with SER change with multivariate linear regression analysis. The results showed no effect on SER change among sex, baseline SER, and baseline AL (*P* = 0.949, 0.671, and 0.800, respectively), whereas age at intervention had a significant effect on SER change (*P* = 0.022). Among the treatment modalities, DIMS + ATP and DIMS had a significant effect on SER change compared to SV (DIMS + ATP, β =  − 0.612, *P* < 0.001, DIMS, β =  − 0. 321, *P* = 0.020).

**Table 3:** Factors associated with changes in AL and SER during the 1-year follow-up using multiple linear regression.

Group	AL1y			SER1y		
	β	95% CI	P value	β	95% CI	P value
DIMS + ATP vs. SV	− 0.262	(− 0.370, − 0.154)	0.000	− 0.612	(− 0.901, − 0.322)	0.000
DIMS vs. SV	− 0.135	(− 0.236, − 0.035)	0.009	− 0.321	(− 0.591, − 0.052)	0.020
Intervention age	− 0.069	(− 0.113, − 0.024)	0.003	− 0.141	(− 0.260, − 0.021)	0.022
Sex (female vs. male)	0.016	(− 0.077, 0.108)	0.740	0.008	(− 0.241, 0.257)	0.949
Baseline SER	0.004	(− 0.040, 0.048)	0.859	− 0.026	(− 0.145, 0.094)	0.671
Baseline AL	0.007	(− 0.059, 0.073)	0.833	0.023	(− 0.155, 0.200)	0.800

*ATP* atropine, *AL* axial length, *CI* confidence interval, *DIMS* defocus-incorporated multiple segments, *SER* spherical equivalent refraction.

Statistical significance was defined as P < 0.05.

## Discussion

Myopia progression and axial elongation were lower in participants receiving a combination of 0.01% ATP and DIMS than in those receiving DIMS alone and SV alone, indicating an additive effect in the combined treatment. Children treated with a combination of 0.01% ATP and DIMS showed a significant reduction in myopia progression by 46% and axial elongation by 54% over 1 year compared to the group of SV lenses. Children treated with DIMS alone showed a reduction in myopia progression by 21% and axial elongation by 26% over 1 year compared to the SV lenses group. To our knowledge, this study is the first to evaluate the additive effects of DIMS and 0.01% ATP on slowing axial elongation in children with myopia.

In this study, axial elongation over 1 year was dramatically slowed by 0.13 mm in participants treated with a combination of 0.01% ATP and DIMS compared with DIMS alone. This result agrees with Kinoshita et al., who reported an increased treatment effect of 0.10 mm when comparing the combination of ortho-k and 0.01% ATP with ortho-k alone in 1 year^24^. Although 0.01% ATP alone had a weak effect on myopia control, its combination with optical methods can effectively increase the effect of myopia control. A combination of optical methods and 0.01% ATP seems to be a good choice for children with myopia or those with faster myopia progression.

The mechanism of myopia control with 0.01% ATP and DIMS remains unclear. A previous study reported that the DIMS lens uses the technique of peripheral myopia defocusing to control myopia^18^. ATP can increase choroidal thickness to control the growth of ocular AL^25,26^. Moreover, 0.01% ATP can dilate the pupil by approximately 0.71 mm^27^, increasing the MD effect of the DIMS lens. The mechanism of myopia control with 0.01% ATP and DIMS may include optical defocus, drug action, and synergistic effects.

Myopia progression over 1 year was reduced by 26% in participants treated with DIMS lenses only compared with those treated with SV lenses alone (mean difference − 0.28 ± 0.13 D, 95% CI − 0.54 to − 0.01, *P* = 0.043). This effect is lower than the result reported by Lam et al., who observed an enhanced treatment effect slower by 52% for children treated with DIMS compared with those with SV lenses (mean difference − 0.44 ± 0.09 D, 95% CI − 0.73 to − 0.37, *P* < 0.0001)^18^. Although both ethnic groups were Chinese, children in eastern mainland China seem to have myopia earlier and progress faster than children in Hong Kong. Lam et al. reported that myopia progression over 1 year was − 0.55 ± 0.04 D in the SV group^18^, whereas our study found that myopia progression over 1 year was − 1.07 ± 0.64DS in the SV group. It seems that + 3.5 D MD of the DIMS lens was effective in patients with 0.55 D/year myopia progression, while in those with 1.07 D/year myopia progression, higher MD may be required. In addition, Walline et al.^28^ found that treatment with high add power multifocal contact lenses significantly slowed myopia progression more than medium add power lenses and single-vision contact lenses in a 3-year double-masked randomized clinical trial. Thus, increasing the dose of MD might be a way to improve the effect of myopia control.

A limitation of this study was its retrospective nature. The design of the study and control groups cannot be randomized, as the parents actually selected the groups, which may result in confounding factors that affect the results. A randomized clinical trial is warranted to further compare the treatment effects of single and combined modalities. Furthermore, our follow-up time was only 1 year, and the combined longer-term effects require further study.

In conclusion, myopia progression and axial elongation were lower in patients treated with a combination of 0.01% ATP and DIMS. Combination therapy of 0.01% ATP and DIMS is an alternative treatment for myopia control.

## Data Availability

Correspondence and requests for materials should be addressed to Chi-Xin Du. Reprints and permissions information is available at www.nature.com/reprints.
