# Author Correction: Synergistic effects of defocus-incorporated multiple segments and atropine in slowing the progression of myopia

**DOI:** 10.1038/s41598-023-36663-7

**Published:** 2023-06-14

**Authors:** Zhu Huang, Xu-Fei Chen, Ting He, Yun Tang, Chi-Xin Du

**Affiliations:** 1 Department of Ophthalmology, The First Affiliated Hospital, College of Medicine, Zhejiang University, Hangzhou, 310003 China; 2 Department of Ophthalmology, The Fourth Affiliated Hospital, School of Medicine, Zhejiang University, Yiwu, 322000 China

Correction to: *Scientific Reports* https://doi.org/10.1038/s41598-022-25599-z, published online 24 December 2022

The original version of this Article contained an error in the Results section, under the subheading ‘Factors associated with AL and SER changes’, where SER was incorrectly written as AL.

“The results showed no effect on AL change among sex, baseline SER, and baseline AL (*P* = 0.949, 0.671, and 0.800, respectively), whereas age at intervention had a significant effect on AL change (*P* = 0.022). Among the treatment modalities, DIMS + ATP and DIMS had a significant effect on AL change compared to SV (DIMS + ATP, β = − 0.612, *P* < 0.001, DIMS, β = − 0.321, *P* = 0.020).”

now reads:

“The results showed no effect on SER change among sex, baseline SER, and baseline AL (*P* = 0.949, 0.671, and 0.800, respectively), whereas age at intervention had a significant effect on SER change (*P* = 0.022). Among the treatment modalities, DIMS + ATP and DIMS had a significant effect on SER change compared to SV (DIMS + ATP, β = − 0.612, *P* < 0.001, DIMS, β = − 0.321, *P* = 0.020).”

